# Event-based surveillance in the WHO African region: baseline implementation before the COVID-19 pandemic

**DOI:** 10.3389/fpubh.2025.1697663

**Published:** 2026-01-21

**Authors:** Opeayo Ogundiran, George Sie Williams, Etien Koua, Theresa Lee, Tatiana Metcalf, Oluwatosin Wuraola Akande, Jessie Abbate, Ifunanya Nwabueze, Joseph Okeibunor, Abou Salam Gueye

**Affiliations:** 1 World Health Organization, Geneva, Switzerland; 2 WHO Regional Office for Africa, Brazzaville, Republic of Congo; 3 Public Health Agency of Canada, Ottawa, ON, Canada; 4 Geomatys, Montpellier, France

**Keywords:** event-based surveillance, epidemic intelligence, infectious disease surveillance, health facility surveillance, community-based surveillance, media monitoring, WHO African region

## Abstract

Event-based surveillance (EBS) is a critical component of epidemic intelligence, supporting the timely detection of unusual public health events. However, prior to the COVID-19 pandemic, the status of EBS implementation across the WHO African region was not well documented. This study establishes a regional baseline of EBS modalities in place before the pandemic to inform future surveillance system evaluation and strengthening. We conducted a cross-sectional survey across all 47 WHO Member States in the African region between December 2019 and February 2020. A structured questionnaire was disseminated through WHO Country Offices to assess the presence, characteristics, and functionality of EBS modalities—including health facility-based EBS (HEBS), community-based surveillance (CBS), and media monitoring. Quantitative data were analyzed descriptively, while qualitative responses underwent thematic analysis. Thirty-seven countries (78.7%) responded, of which 84% (*n* = 31) had implemented at least one form of EBS. HEBS was the most commonly reported modality (67.6%), followed by CBS (54.1%) and media monitoring (29.7%). Cholera, acute flaccid paralysis, measles, and meningitis were the most frequently detected conditions. While mobile phones were the primary channel for reporting, challenges included resource constraints and infrastructure gaps. Radio was the most widely used media source due to limited access to internet-based tools. Countries widely recognized EBS as a national priority but cited the need for sustainable financing, tools, and trained personnel. By early 2020, most countries in the region had adopted EBS, though implementation varied in scope and maturity. The COVID-19 pandemic catalyzed expansion of many modalities, offering an opportunity to institutionalize gains. Strengthening EBS systems will require sustained investment, intersectoral coordination, and context-appropriate tools aligned with national capacities.

## Introduction

The African continent experiences a disproportionately high burden of infectious disease outbreaks, often compounded by fragile health systems and limited surveillance capacity (1–3). These systemic weaknesses have historically delayed the detection and response to public health threats, with devastating consequences—as seen during the 2014–2016 Ebola virus disease (EVD) outbreak in West Africa and more recently, the COVID-19 pandemic (4, 5).

To address these challenges, the International Health Regulations (IHR 2005) require that Member States establish core capacities for the timely surveillance, detection, and response to public health emergencies (6, 7). In the WHO African region, the Integrated Disease Surveillance and Response (IDSR) strategy—launched in 1998—serves as the primary framework for national surveillance systems (8). The IDSR strategy incorporates both indicator-based surveillance (IBS), involving regular structured data reporting from health facilities, and event-based surveillance (EBS), which focuses on the rapid identification of unstructured signals or unusual events that may indicate public health threats (9).

Event-based surveillance is defined as the organized collection, monitoring, assessment, and interpretation of primarily unstructured information that may signal an acute public health risk (10). EBS sources include communities, schools, environmental and animal health sectors, and both traditional and digital media. Implementation occurs through several modalities—commonly community-based surveillance (CBS), health facility-based EBS (HEBS), public hotlines, and media monitoring (10).

Despite its strategic importance, evaluations of IDSR implementation before 2020 found that most countries remained heavily reliant on IBS, with limited mechanisms to capture atypical events occurring outside the formal health system (9). To address this gap, the Africa Centres for Disease Control and Prevention (Africa CDC), in collaboration with WHO and Member States, introduced the Framework for Event-Based Surveillance in Africa in 2018 to guide systematic EBS implementation across the continent.

Although the utility of EBS is increasingly recognized, its application prior to the COVID-19 pandemic remained fragmented and uneven across the African region (10). Nevertheless, EBS demonstrated clear value in several high-priority settings. It supported preparedness for the 10th EVD outbreak in the Democratic Republic of the Congo (DRC) in 2019 (11), enhanced acute flaccid paralysis (AFP) detection through mobile-based CBS platforms during polio eradication efforts (12), and enabled early alerts for COVID-19 and subsequent EVD outbreaks in Guinea and eastern DRC (13).

Despite these successes, system-level gaps persisted. As of March 2019, only 38% of the 40 countries in the African region that underwent Joint External Evaluations (JEEs) were rated as having adequate disease surveillance capacity (14, 15). Moreover, the median delay between symptom onset and outbreak notification for reported events between 2017 and 2019 was up to eight days—highlighting the need for more responsive detection mechanisms (16).

Significant investments in surveillance infrastructure were made during the COVID-19 pandemic, including the rapid expansion of national hotlines and increased adoption of digital tools such as the Epidemic Intelligence from Open Sources (EIOS) platform (17). However, this study deliberately focuses on the pre-COVID-19 baseline to provide a benchmark for understanding the extent to which countries had already institutionalized EBS capacities before external shocks prompted emergency adaptations. Establishing this baseline is essential for disentangling what was developed through sustained national efforts versus what emerged from urgent, externally driven pandemic responses. It also helps identify foundational strengths and vulnerabilities that shaped the region’s initial COVID-19 response capacity.

By establishing this baseline, we aim to provide a reference point for evaluating post-pandemic progress and guiding future strategies to strengthen epidemic intelligence in the WHO African region.

## Methods

### Study design

We conducted a descriptive, cross-sectional study using a mixed-methods approach to characterize the implementation of event-based surveillance (EBS) systems in the 47 Member States of the WHO African region prior to the onset of the COVID-19 pandemic.

### Definitions

For the purposes of this study, EBS was defined as the organized collection, monitoring, assessment, and interpretation of primarily unstructured, *ad hoc* information regarding public health events or risks that may represent an acute threat to human health (18–20). We categorized EBS systems into three modalities:

- Health Facility-based EBS (HEBS): Immediate reporting of suspected health events by healthcare workers based on clinical encounters or indirect information about unusual occurrences within or around health facilities (10).
- Community-based surveillance (CBS): Systematic detection and reporting of events of public health significance by community members or local informants (21).
- Media monitoring: Active and continuous review of media sources - such as radio, newspapers, online news, social media - to identify signals that may indicate public health threats (22).

### Data collection

Data were collected using a structured online questionnaire disseminated to all 47 Member States between 9 December 2019 and 5 February 2020. The survey was available in English and French and was administered to personnel responsible for disease prevention and control, including Diseas0e Prevention and Control (DPC) officers, Health Emergency Information and Risk Assessment (HIM) focal points, Infectious Hazard Management (IHM) officers, and WHO Health Emergency (WHE) cluster leads at the WHO Country Offices.

Participants were contacted in advance via email and briefed on the objectives of the study. The questionnaire collected information on the presence and characteristics of EBS systems, including year of establishment, diseases reported, tools used for reporting, geographic coverage, and the structure of alert management teams.

The survey was administered using Google Forms. Follow-up reminders and direct outreach were conducted to maximize the response rate and minimize missing data.

### Data analysis

Quantitative data were analyzed descriptively to assess the geographic distribution, frequency, and features of the different EBS modalities. Key variables analyzed included modality type, geographic scope, year of establishment, diseases reported, reporting tools, and the administrative level of alert management systems.

Qualitative data from open-ended survey responses were analyzed thematically using an inductive approach (23). Two independent coders reviewed the data, generated preliminary codes, and grouped recurring concepts into higher-level themes. Discrepancies were resolved through discussion, and final themes were refined to reflect respondent perspectives on EBS implementation.

All statistical analyses were conducted using R version 4.0.4 (24), and geospatial mapping was performed with ArcGIS Pro version 2.1.0 (25).

## Results

**Figure 1:**
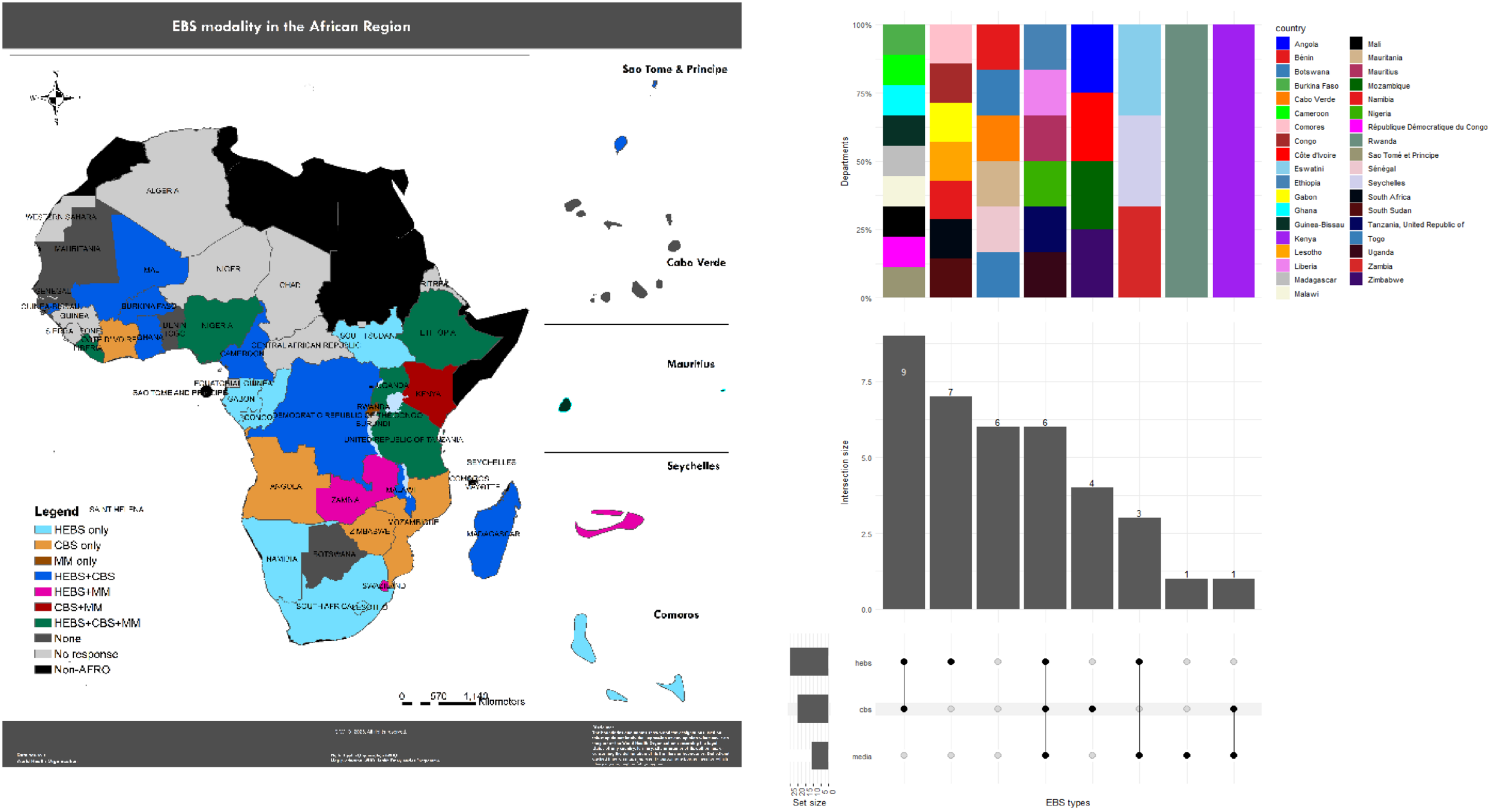
Adoption of event-based surveillance (EBS) modalities in the WHO African region, 9 December 2019–5 February 2020 (*n* = 37/47 Member States). Bars/map indicate countries reporting health-facility EBS (HEBS; 25/37, 67.6%), community-based surveillance (CBS; 20/37, 54.1%), and media monitoring (11/37, 29.7%); six countries (Ethiopia, Liberia, Mauritius, Nigeria, Tanzania, Uganda) reported all three. Data are self-reported from the survey and reflect respondents only. EBS, event-based surveillance; HEBS, health-facility EBS; CBS, community-based surveillance; MM, media monitoring.

A total of 37 out of 47 WHO Member States in the African region responded to the survey, yielding a response rate of 78.7%. Of these, 31 Member States (84%) had implemented at least one form of event-based surveillance (EBS) prior to the onset of the COVID-19 pandemic. Health facility-based EBS (HEBS) was the most widely adopted modality, reported in 25 Member States (67.6%), followed by community-based surveillance (CBS) in 20 Member States (54.1%) and media monitoring in 11 Member States (29.7%). Six Member States —Ethiopia, Liberia, Mauritius, Nigeria, Tanzania, and Uganda—had implemented all three modalities (Figure 1).

### Health facility-based EBS (HEBS)

HEBS systems were operational in 25 Member States, with 22 of them reporting national-level coverage. The remaining three had implemented HEBS in selected districts. In terms of maturity, 56% of Member States had established HEBS systems one to three years before the survey, 24% had implemented them within the preceding year, and 20% had systems that had been in place for more than three years.

Cholera was the most frequently reported condition monitored through HEBS, cited by 88% of Member States, followed by acute flaccid paralysis (AFP) and meningitis (both at 84%), measles (80%), and viral haemorrhagic fevers (76%). The most common tool for reporting HEBS alerts was the mobile phone, used in 80% of Member States. Other tools included software applications (40%), email (36%), hotlines (28%), and paper-based forms (24%).

National-level alert management teams were in place in 76% of Member States implementing HEBS. Additionally, 40% reported having district-level teams, and 36% had provincial-level teams, suggesting a moderately decentralized capacity for signal verification and response coordination.

### Community based surveillance (CBS)

CBS systems were reported in 20 Member States, with half of them having implemented CBS nationally, while the remainder restricted it to selected districts or provinces. Twelve Member States had CBS systems that had been in operation for more than three years, six had systems in place for one to three years, and two had introduced CBS within the previous 12 months.

The conditions most commonly monitored through CBS were similar to those covered by HEBS. Eighty percent of Member States reported surveillance for AFP and cholera, 75% for measles, 65% for meningitis, and 55% for yellow fever. As with HEBS, phone calls were the primary means of alert transmission, used in 75% of Member States. Software applications (30%), hotlines (15%), and email (15%) were also reported. Additionally, 35% of Member States used other methods such as social media platforms, verbal reports to health workers, and paper-based tools.

CBS systems were more frequently supported by decentralized alert management structures. Seventy percent of Member States reported having district-level alert teams, 60% had teams at the national level, and 30% had provincial-level teams.

### Media monitoring

Media monitoring was the least implemented modality, reported by only 11 Member States. All systems were implemented at the national level. Most had been in operation for more than three years (55%), while others were established within the previous year (27%) or between one and three years prior (18%).

Radio was used universally as a source for media monitoring, reflecting its wide accessibility across the region. Other commonly used sources included social media platforms (91%), newspapers (73%), and online news websites (73%). Television was only mentioned by Uganda.

In terms of tools, ProMED was the most frequently cited (55%), followed by Epidemic Intelligence from Open Sources (EIOS) at 27%, and HealthMap and GPHIN, each used by one Member State. Media monitoring was applied to a broad range of public health hazards. Seventy-two percent of Member States using this modality reported monitoring for AFP, cholera, measles, meningitis, dengue, influenza-like illnesses, viral haemorrhagic fevers, yellow fever, and chemical or natural hazards. Monitoring frequency also varied, with 72% of Member States conducting daily media reviews, while the remainder reported irregular monitoring.

**Figure 2:**
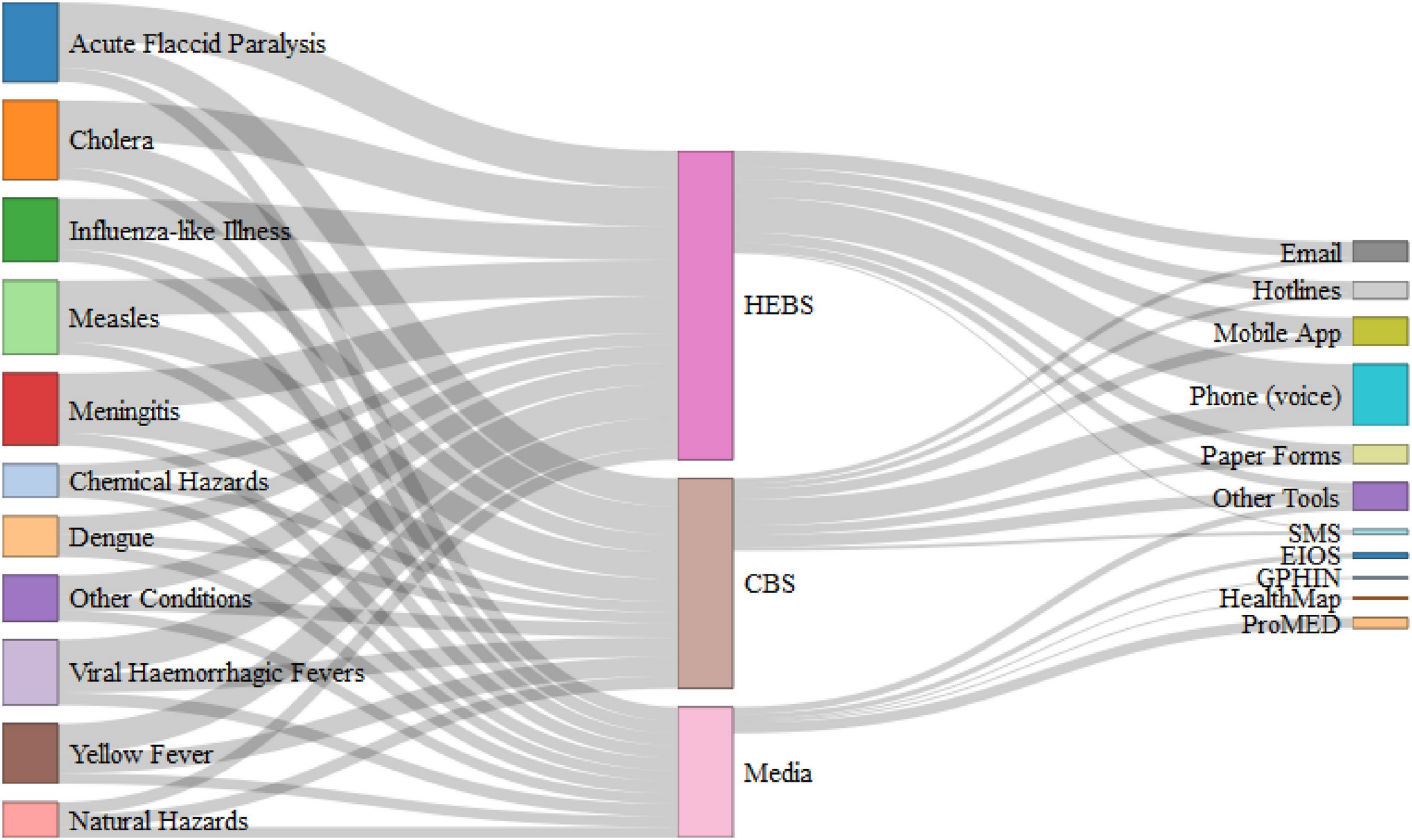
Alluvial diagram linking conditions monitored, EBS modalities, and reporting tools/platforms among 37 responding WHO African region Member States (surveyed 9 December 2019–5 February 2020). Flow width reflects the number of countries reporting each linkage. Countries could select multiple conditions and tools; totals therefore exceed the number of respondents.

A consolidated overview of EBS implementation across countries—including the extent of modality adoption, administrative levels of alert verification teams, and duration of implementation—is provided in Supplementary Figure 1, while Figure 2 illustrates the relationships between conditions monitored, EBS modalities, and reporting tools, highlighting the diversity and overlap of surveillance pathways across the region.

### Thematic analysis of open-ended responses

Twenty-nine Member States (78% of respondents) provided open-ended responses, offering valuable perspectives on the implementation and utility of EBS. A recurring theme was the recognition of EBS as a critical complement to routine indicator-based surveillance. Many Member States described efforts to align EBS with their national IDSR strategies and noted that expansion of EBS was a growing priority.

Several Member States indicated plans to scale up EBS modalities, particularly CBS and media monitoring, although these plans often depended on the availability of external funding or technical assistance. Respondents also highlighted persistent operational challenges. Human resource limitations, including high staff turnover and insufficient training, were frequently cited. Infrastructure gaps—such as lack of mobile phones or digital tools among frontline health workers and community volunteers—also hampered timely reporting. As one respondent explained, “*Most of the community health workers do not have mobile phones to support timely reporting of alerts or events*,” while another noted that “*facilities are not equipped with devices to effectively report on this platform.”*

## Discussion

This study provides a region-wide snapshot of event-based surveillance (EBS) implementation in the WHO African region just prior to the COVID-19 pandemic. While the majority of countries reported having at least one form of EBS in place, our findings highlight significant differences in the maturity and structure of implementation across modalities, administrative levels, and settings. These differences underscore both the value of EBS and the foundational challenges that must be addressed to achieve comprehensive, functional, and sustainable epidemic intelligence systems in the region (26–28, 48).

The COVID-19 pandemic catalyzed rapid expansion of several EBS modalities in the region, including the introduction of national hotlines, wider use of digital platforms such as the Epidemic Intelligence from Open Sources (EIOS), and the establishment of dedicated epidemic intelligence teams (29). Although these developments occurred after the period under study, the pre-pandemic baseline established here is essential for assessing how much of the current capacity was built on existing foundations versus emergent responses to crisis. The results also offer insight into where foundational investments were already being made and where gaps were likely to constrain early response.

### EBS modalities and maturity

The three primary EBS modalities examined reflect different entry points for early detection of public health threats. Their varying maturity levels across countries reveal important lessons about implementation pathways and feasibility.

HEBS appeared to be the most widely established modality, benefiting from its integration with routine indicator-based surveillance and use of existing health facility infrastructure. Its relatively higher adoption likely reflects the ease with which HEBS can be embedded into formal reporting workflows and the presence of trained personnel in clinical settings (9). The existence of alert management teams at national and subnational levels further supports rapid signal triage, helping to shorten the delay between detection and response. HEBS can thus be considered a practical and scalable starting point for countries seeking to expand EBS capacity, particularly in systems with well-established facility-based surveillance.

In contrast, CBS served a critical complementary role, especially in detecting unusual events in communities with limited access to health services. In many settings, CBS systems evolved independently of national EBS frameworks, often originating from vertical programs such as polio eradication or humanitarian health interventions (15, 30). While this organic growth allowed CBS to fill critical detection gaps in hard-to-reach areas, it also led to fragmented implementation and varying levels of integration into broader health systems. Sustainability remains a central challenge: frequent staff turnover, insufficient training, reliance on volunteer health workers, and lack of standardized tools all limit CBS performance in many contexts (31). Yet, when properly supported and institutionalized, CBS offers substantial value for real-time surveillance in rural and underserved populations.

Media monitoring, the least mature of the three modalities, was constrained by systemic factors such as limited internet access, low digital literacy among surveillance personnel, and underinvestment in open-source intelligence infrastructure (32). Despite these constraints, media monitoring presents a major opportunity to broaden surveillance reach and detect threats that may not emerge through traditional channels. Especially as countries expand their use of platforms like EIOS and build capacity in digital epidemic intelligence, media monitoring has the potential to complement both HEBS and CBS by capturing events reported in local media, social networks, or online news outlets (13).

The observed differences in modality adoption and maturity reflect the broader diversity of health system capacity, implementation strategy, and funding mechanisms across the region. Building a robust EBS system requires not only technical guidance but also adaptive implementation approaches that match the structural realities and priorities of each country.

### Diseases monitored through EBS

The types of conditions most commonly reported through EBS reflect both the health priorities of countries and the functional characteristics of EBS modalities. Diseases such as cholera and acute flaccid paralysis (AFP) were consistently among the most frequently flagged, owing to a combination of programmatic, structural, and epidemiological factors.

Cholera remains endemic in many parts of the African region, particularly in settings with poor access to safe water, sanitation, and hygiene infrastructure (33–35, 49–51). This persistent risk, coupled with periodic large-scale outbreaks, has led to heightened awareness at both community and health system levels. Additionally, dedicated surveillance initiatives—such as the African Cholera Surveillance Network (Africhol) and the Global Task Force on Cholera Control (GTFCC) — have reinforced early detection mechanisms, particularly in hotspot areas (33, 34, 36). These factors increase the likelihood of cholera being recognized and reported as a public health event.

Similarly, AFP has long been a surveillance priority in the context of the polio eradication initiative (35). For decades, countries have received technical and financial support to maintain sensitive AFP detection systems as part of certification processes (37, 38). The emphasis on case detection—supported by well-established reporting chains and performance monitoring indicators—has strengthened frontline capacities to recognize and report AFP syndromes quickly and accurately.

Both cholera and AFP are also relatively straightforward to detect syndromically. In the case of cholera, the presentation of acute watery diarrhoea—often occurring in clusters or during seasonal peaks—is widely recognized in endemic communities. AFP presents as a sudden and visible neurological deficit, which is both alarming and distinctive, even to non-clinical observers. These characteristics make such conditions well suited for detection through EBS modalities that rely on preliminary clinical impressions or community informants without the benefit of laboratory confirmation.

In contrast, diseases with non-specific or common symptoms—such as febrile illnesses or respiratory infections—are significantly more challenging to detect through EBS. Their clinical presentation often overlaps with a wide range of endemic conditions, including malaria, influenza, and other viral illnesses, which may not trigger concern unless they appear in large numbers or are associated with severe outcomes. As a result, signals related to these syndromes may go unnoticed, unreported, or deprioritized, especially in communities where such symptoms are considered routine. This highlights an important limitation of EBS: its effectiveness is shaped not only by system design but also by the recognizability and perceived significance of a condition’s clinical presentation.

Hazards outside the infectious disease domain, such as chemical spills, environmental threats, or natural disasters, were rarely reported through EBS. This gap likely reflects both structural fragmentation between health and other sectors (e.g., environment, civil protection, animal health) and a limited awareness or mandate among health surveillance personnel to report such events. Enhancing all-hazard surveillance will require multisectoral coordination, joint planning, and expanded training to support a broader interpretation of what constitutes a “public health event.”

### Tools used for EBS in the WHO African region

The tools used to facilitate EBS reporting varied significantly across countries and modalities, reflecting both innovation and inequity in access to surveillance-enabling technologies. Mobile phones emerged as the most commonly used tool for EBS reporting, particularly in health facility-based and community-based surveillance systems. Their widespread use was largely due to the accessibility of mobile networks in many parts of the region, combined with the relative simplicity and speed of voice calls or text messaging for transmitting alerts (39). However, even this seemingly universal tool exposed major infrastructure gaps. In several countries, frontline health workers—especially community volunteers—lacked consistent access to mobile phones, electricity, or credit for calls and data, limiting their ability to report in a timely and reliable manner (40). Despite the promise of mobile tools, verbal reporting remained prevalent, particularly in CBS. This often requires district surveillance staff to physically visit communities to gather information, which poses a risk of data loss and delay. Transitioning to digital tools must be coupled with efforts to build digital literacy and ensure connectivity in remote settings.

Media monitoring tools revealed similar disparities. Traditional media sources—especially radio—were widely used for event detection, reflecting their broad reach in rural and low-connectivity settings (41). In contrast, more sophisticated platforms such as the Epidemic Intelligence from Open Sources (EIOS), ProMED, HealthMap, and GPHIN were underutilized at the time of this study. Their limited adoption can be attributed to several factors, including inadequate technical training, language barriers, and a lack of institutional frameworks for using open-source information as part of routine surveillance (42).

Nevertheless, the value of digital epidemic intelligence tools became more widely recognized during the COVID-19 pandemic. As countries confronted the need for rapid situational awareness, there was a significant uptick in the use of EIOS and similar platforms, alongside investments in training and structured epidemic intelligence functions (17). These developments point to a growing opportunity to formally integrate open-source digital tools into national EBS systems. However, sustained progress will require embedding these platforms into standard workflows, assigning dedicated personnel, and ensuring ongoing institutional capacity building.

Public health hotlines represent another important tool for event detection and public engagement. Prior to the COVID-19 pandemic, hotlines were not widely incorporated into EBS systems. Their use was often limited to *ad hoc* risk communication efforts or small-scale initiatives. However, during the pandemic, many countries rapidly established national hotlines to triage alerts, answer public queries, and link suspected cases with response services (43). This rapid scale-up demonstrated the region’s capacity to deploy responsive surveillance infrastructure under pressure. Going forward, countries should explore how these hotline systems can be sustained and expanded to support multi-hazard detection, integrate community engagement, and serve as part of a broader epidemic intelligence architecture.

Ultimately, the diversity of tools used—and the gaps in their availability and integration—highlight the need for a tiered, adaptable approach to EBS technology. While sophisticated platforms offer powerful capabilities, simpler tools like mobile phones and radios continue to play an indispensable role in surveillance, particularly in resource-limited settings. Investing in connectivity, interoperability, and training will be critical to ensuring that all countries in the region can benefit from the full spectrum of technological options available for epidemic intelligence.

### Administrative coverage and alert management

The effectiveness of EBS depends not only on the detection of signals, but also on the capacity to verify, triage, and respond in a timely and coordinated manner. These functions are typically carried out by alert management teams, whose presence and distribution across administrative levels play a critical role in ensuring that potential public health events are appropriately handled.

Across the WHO African region, the organization of alert management structures reflected broader differences in health system governance and decentralization. HEBS, which tends to operate within the formal health sector, was more likely to benefit from established administrative hierarchies. In countries where national and subnational surveillance units were already functional, alert verification processes could be integrated into existing workflows. This allowed for clearer escalation pathways, more consistent documentation of signals, and better coordination of public health responses (44).

In contrast, community-based surveillance (CBS) often suffered from weaker administrative integration. While CBS played a vital role in detecting signals at the periphery of the health system, many community-based systems lacked clearly defined structures for alert verification. In several cases, community health workers reported signals directly to local facilities or district officers, but follow-up depended heavily on the presence of motivated individuals, local resources, or external support. Where alert management teams existed at district level, CBS verification was more efficient. However, in the absence of structured subnational teams, community-generated signals could be delayed, deprioritized, or lost altogether. This underscores the need to strengthen the vertical linkage between community-level detection and formal public health action (45).

Media monitoring presented a distinct challenge. Most media monitoring systems were centralized at the national level, with dedicated surveillance or communications units responsible for scanning sources, identifying relevant events, and initiating verification. While this model ensured consistency and centralized oversight, it also introduced vulnerabilities. The reliance on a small number of individuals or units limited scalability, and the absence of subnational media scanning reduced sensitivity to local events—particularly those reported in local languages or community-based outlets. Countries with large geographic areas or devolved governance structures may benefit from decentralizing aspects of media monitoring, such as regional scanning or initial triage, to improve responsiveness (27).

The experience of the COVID-19 pandemic highlighted both the potential and the fragility of alert management systems. In the early months of 2020, countries across the region rapidly established or expanded alert verification teams, many of which were linked to national hotlines or emergency operations centres. These teams often included newly appointed staff, digital platforms for recording alerts, and formal escalation protocols—demonstrating the ability to build surveillance infrastructure quickly under pressure (46). In several countries, the pandemic response also catalyzed the development of subnational verification teams, marking a significant step toward decentralization.

These emergency-driven achievements, however, were often supported by temporary funding and external technical assistance (47). Sustaining them will require formal incorporation into national surveillance strategies, budget lines for dedicated personnel and tools, and continuous training to adapt to evolving threats. Moreover, alert management should not be viewed as an isolated function, but as part of a broader epidemic intelligence cycle that links detection to response.

As countries continue to invest in EBS, strengthening alert management structures—particularly at the subnational level—should be a priority. Doing so will not only improve timeliness and coordination but will also increase trust in the system by ensuring that reported signals lead to visible action.

## Conclusion

By early 2020, the majority of countries in the WHO African region had adopted at least one event-based surveillance (EBS) modality, reflecting growing recognition of its role in improving epidemic intelligence. However, the scope and maturity of these systems varied considerably, with many countries relying on fragmented or partially institutionalized approaches. This study provides a unique pre-pandemic baseline of EBS implementation across the region, offering critical insights into the foundations upon which subsequent COVID-19 surveillance innovations were built.

The pandemic demonstrated both the urgency and feasibility of scaling EBS modalities—including national hotlines, digital platforms, and alert verification teams—when supported by political commitment and external investment. Yet, many of these gains were reactive and remain at risk of erosion without deliberate efforts to institutionalize them.

Strengthening EBS systems going forward will require sustained investment in community-based and digital surveillance, formal integration of alert management into national structures, and multisectoral coordination to support all-hazard detection. Leveraging the momentum generated during the pandemic to build resilient, integrated, and context-appropriate EBS systems is essential to ensuring early detection and response to future public health threats in the African region.

## Data Availability

The raw data supporting the conclusions of this article will be made available by the authors, without undue reservation.
